# Supplementary material for: Chromothripsis during telomere crisis is independent of NHEJ, and consistent with a replicative origin
Source: Genome Res. 2019 May;29(5):737–49. doi: 10.1101/gr.240705.118 (PMC6499312; doi:10.1101/gr.240705.118)
Supplement: Supplemental Material [file supp_gr.240705.118_Supplemental_file_1.zip › contigs/annotated_contigs/DB115/contig.2.DB115_length_474_mean_cov_6.32489451477.docx]

**DB115_length_474_mean_cov_6.32489451477**

```
ATCAGTGGCAGAGCTGGGACGAAACCCCAACGTCTCCTGAGGCCCAGCCATGAGCTAGTTTCCCATTCTACCACATCCTCACTTTCATG
 >chr3:66540179-66540413 + E=3e-129
ACTTTAAGAAAATTAATTTTTTCCTCTCAGGTGAATCATTTTTATGCCTCTTTGGGCAAGCACCCCTTTAAGAAACTCCATGAAAACGT

GGTCTCCTTTCTGTCCAGAAAGACATTCTGAGCACCACGACTTCACATAAAGTTTC|AAATATTTTATA|AGCCCCTCACAGACTTATA
                                                                     >chr3:65967181-659
AAATATTATATGAGAGACTGTATTAGATATATGTGGTTAGTACTTCATATGGTTTGGCTGTGTCCTCACCCTTCTCATCTTGAATTGTA
67409 + E=1e-125
GCTCCCATAATTCCCACGTGTTGTGGGAGGCACCCGGTGGGAGATAACTGAATCATGGGGGTGGTTTCCCCCATATTGCTCTGGTGGTA

GTGAATAAGTCTCACAAGAACTGATGGTTTT
```
